# Pediatric Cutaneous Leishmaniasis in an Endemic Region in Turkey: A Retrospective Analysis of 8786 Cases during 1998-2014

**DOI:** 10.1371/journal.pntd.0004835

**Published:** 2016-07-14

**Authors:** Mustafa Aksoy, Nebiye Doni, Hatice Uce Ozkul, Yavuz Yesilova, Nurittin Ardic, Abdullah Yesilova, Jennifer Ahn-Jarvis, Steve Oghumu, Cesar Terrazas, Abhay R. Satoskar

**Affiliations:** 1 Department of Dermatology, Harran University School of Medicine, Sanliurfa, Turkey; 2 Department of Microbiology, Harran University School of Medicine, Sanliurfa, Turkey; 3 Department of Dermatology, Yuzuncu Yıl University School of Medicine, Van, Turkey; 4 Ministry of Health, Health Sciences University, Van Training and Research Hospital, Dermatology Clinic, Van, Turkey; 5 Department of Microbiology, Gulhane Military Medical Academy, Ankara, Turkey; 6 Department of Biostatistics, Yuzuncu Yıl University School of Medicine, Van, Turkey; 7 Biosciences, College of Dentistry, Ohio State University, Columbus, Ohio, United States of America; 8 Environmental Health Sciences, College of Public Health, Ohio State University, Columbus, Ohio, United States of America; 9 Department of Pathology, Ohio State University Medical Center, Columbus, Ohio, United States of America; Fundaçao Oswaldo Cruz, BRAZIL

## Abstract

**Background:**

Cutaneous leishmaniasis (CL) is a major public health concern in Turkey and Sanliurfa represents the most endemic city in Turkey. Although children are most commonly affected by CL, detailed studies of pediatric CL in Turkey are lacking.

**Methodology/Principal Findings:**

In this report we retrospectively evaluated clinical and epidemiological data of 8786 pediatric CL cases, and how children respond to antimonial therapy. CL was observed most frequently in children between 6–10 years old. Interestingly this group showed shorter duration of disease and smaller lesions compared to 0–5 year and 11–15 year old groups. Females were more affected in all groups. Lesion localization and types varied among groups, with 0–5 year old presenting head/neck and mucosal lesions, and more often suffered from recidivans type, this could be associated to the longest duration of the disease in this group. Eleven-15 year old group showed fewer lesions in the head/neck but more generalized lesions. Evaluation of treatment response revealed that intra-lesional treatment was preferred over intramuscular treatment. However, 0–5 year old received intramuscular treatment more often than the other groups. Furthermore, the majority of 0–5 year old group which received intra-lesional treatment did not received subsequent intra-lesional cycles, as did children in the range of 6–15 years old.

**Conclusions/Significance:**

We report an increase in pediatric CL patients within the last four years. Analysis of pediatric CL patients by age revealed significant differences in CL progression. The data suggest that children between 0–5 years old responded better than other groups to intralesional treatment, since they received more often a single cycle of IL treatment, although follow up observation is required since they were more prone to develop recidivans. Eleven-15 year old patients comprise the largest percentage of patients receiving two or three cycles of intralesional treatment, suggesting that this group did not respond efficiently to intralesional treatment and highlighting the need for more effective therapeutic strategies against CL.

## Author Summary

In this study, 8786 pediatric (0-15years old) cutaneous leishmaniasis (PCL) patients were retrospectively evaluated for the epidemiological and clinical characteristics. From the records of PCL patients, we focused on the patients' age, gender, lesion type and location, diameter, number and duration of the lesions, and the treatment given. Of the patients, 4050 (46·1%) were males and 4736 (53·90%) were females, with a mean age of 7·52 years (range 0–15 years). Mean lesion diameter was 12·77±0·11 mm (range 1-100mm), while the average lesion duration and the number of lesions was 8·58±0·21 weeks and 1·76±0·01, respectively. The area most involved in the lesions of PCL patients was the head and neck region at a rate of 32·55% (n = 2230). The most (78·79%) of the PCL patients who received intralesional pentavalent antimonial therapy responded to the first cycle of treatment. It was observed that the PCL patients who applied systemic pentavalent antimonial therapy, resistant to intralesional pentavalent antimonial therapy, were completely recovered. We believe that this study will contribute to clinicians to evaluate the clinical features and therapy choice of pediatric CL patients.

## Introduction

Cutaneous leishmaniasis (CL) is a skin infection caused by various species of the parasite *Leishmania*, and is spread by the bite of an infected female *Phlebotomine* sand flies [1,2]. Worldwide, there are 1·5–2 million new CL cases annually and it is estimated that 350 million people are at risk of infection [2]. The number of CL cases has significantly increased in Turkey in recent years representing a major public health problem [3]. In the Old World, CL is often caused by *L*. *tropica* and *L*. *major* [3–8]. Skin lesions caused by CL usually heal spontaneously or become chronic [9]. The vast majority of CL cases are seen in childhood. In epidemiological studies, it has been shown that children are at greater risk than adults[10–14].

Turkey is an endemic country for CL and the main causative agent is *L*. *tropica*. However, CL caused by *L*. *infantum* and *L*. *major* has also been reported especially from East part of the Mediterranean Region [15,16]. Within the last two years of this study (2012–2014), an outbreak of leishmaniasis occurred in Turkey, which was associated with the Syrian civil war and has severely affected local efforts at controlling the spread of disease [16,17]. CL affects mainly the Southeastern Anatolia region of Turkey, which is close to the border with Syria with about 61% of CL cases, and Sanliurfa represents the most endemic city in this region [8]. Previous studies have shown that 0–19 year old patients represent 60–70% of the total population in Turkey infected with CL, emphasizing the importance of evaluating this population in more detail. However, within this population, studies characterizing age and gender based incidence, as well as age-bias correlations in response to various forms and cycles of treatment have not been fully examined. These data are vital in the reevaluation of prevention and treatment strategies against CL, owing to its ever growing incidence in this region. In this study we examined the epidemiological and clinical characteristics of pediatric (0–15 years old) cutaneous leishmaniasis patients (PCL) over a 16-year period in the province of Sanliurfa, Turkey.

## Materials and Methods

### Data Collection

This study retrospectively evaluated 8786 PCL patients aged 0–15 years, who were registered at Harran University Medical Faculty Dermatology Department and the Public Health Oriental Boil Centre between 1998 and May 2014. Diagnosis of PCL was determined based on the patient’s clinical appearance, laboratory tests (positive demonstration of *Leishmania* parasites (amastigotes) in skin smears and evaluation of histopathology results). From the records of PCL patients at these centers we evaluated patient age, gender, lesion type (ulcer, papule, nodule, recidivans), lesion location (head-neck, trunk, upper extremity, lower extremity, mucosa, generalized), lesion diameter (mm), number of lesions, duration of lesions (weeks), and the treatment given (intralesional and systemic pentavalent antimonial treatment).

### Ethics Statement

The study was approved by the local Clinical Research Ethics Committee (document date: May 29, 2014 and document number: 74059997.050.01.04/75). Patient medical data were anonymized in this study.

### PCL Treatment

Treatment with intralesional pentavalent antimonial (Glucantime) therapy was administered twice a week for 4 weeks (a total of 8 injections) to all patients diagnosed with PCL disease at the Dermatology Clinic of Harran University Medical Faculty and the Oriental Boil Centre of the Sanliurfa Public Health Directorate.

At the 20-day assessment period after treatment with intralesional pentavalent antimonial therapy, the PCL patients who had not recovered were administered a second and third treatment cycle with a similar regimen as in the first treatment cycle (8 injections twice weekly for 4 weeks). PCL patients with more than 5 lesions who did not recover after intralesional pentavalent antimonial injection, those with lesions larger than 5 cm, those with genital and oral mucosa involvement and those with lesions in cartilage tissue [2,18] were administered systemic pentavalent antimonial therapy for 20 days at 10-20mg/kg/day.

### Statistical Evaluation

Statistical evaluation was performed using SPSS software Version 21 (IBM Inc., Chicago, Il, USA) and *p-values ≤* 0.05 were considered to be significant. Median and interquartile range was used to report clinical characteristics and frequency tables were used to express epidemiological findings. Lesion diameter, duration of disease, and number of lesions data was not normally distributed (Kolmogorov Smirnov test). Therefore, the Kruskal-Wallis test (corrected for ties) for nonparametric data was used to analyze overall differences with respect to age, gender, lesion localization, lesion type, and treatment. When significant differences were found, Dunn-Bonferroni test was used to make pairwise comparisons [19]. Kendall’s tau b correlation was used to discriminate significant associations between sequential treatment with IL and lesion size or number of lesions. Independent t-test was used to obtain the differences between the groups according to duration, number of lesion and lesion size.

## Results

### General Characteristics of the Pediatric Cutaneous Leishmaniasis Patients

The study comprised a total of 8786 pediatric patients of which 4050 (46·1%) were males and 4736 (53·90%) were females, with a mean age of 7·52 years (range 0–15 years). The majority of the PCL patients consulted a doctor within 0–6 weeks of the onset of disease. Mean lesion diameter was determined as 12·77±0·11 mm (range 1–100 mm), while the average lesion duration was 8·58±0·21 weeks (range, 1–815 weeks), although 66·03% of the pediatric patients resolved the infection within 6 weeks. The number of lesions was 1·76±0·01 mm (range 1–37 mm), with 91·20% of the patients showing 1–3 lesions, while 0.83% presented more than 8 lesions (Table 1).

**Table 1:** General features of patients with pediatric cutaneous leishmaniasis in Sanliurfa, Turkey. (1998-May 2014)

	n	Percentage	Average
**Age (years)**			7.52
0–5	3098	35.26	
6–10	3464	39.43	
11–15	2194	25.31	
**Gender**			
Male	4050	46.1	
Female	4736	53.9	
**Duration (weeks)**			8.58±0.21
0–6	5749	66.03	
6–12	2211	25.39	
>12	747	8.58	
**Lesion diameter**			12.77±0.11
**No. Lesions**			1.76±0.01
1	5337	61.23	
2	1831	21.00	
3	781	8. 96	
4	332	3.80	
5	185	2.12	
6	125	1.43	
7	52	0.59	
>8	73	0.83	

### Distribution of Age, Duration of Lesions and Number of Lesions of the Pediatric Cutaneous Leishmaniasis Patients

To better understand the epidemiological and clinical characteristics of PCL patients in this region, we divided the patients into three age groups: 0–5, 6–10 and 11–15 years old. We found that 6–10 year old patients were the most infected group (Table 1). We investigated whether there were differences in the frequencies of infected males and females among groups. Interestingly the frequency of infected females was higher than males in all age groups. We further observed that 6–10 year-old patients resolved their infection faster and displayed smaller lesion sizes compared with the other groups. The 0–5 year old group showed the longest duration of the disease. No difference in lesion size was found between 0–5 and 11–15 year old groups. The mean of number of lesions was also similar among these 2 age groups (Table 2).

**Table 2:** Distribution of age, gender, duration (weeks), number of lesions, and lesion size (mm) of pediatric cutaenous leishmaniasis patients. Total number and frequencies of patients were divided by gender and group age. Duration of the disease and lesion size were analyzed using Kruskal-Wallis test followed by posthoc comparison with Dunn-Boneferroni test.

Age	Male	Female	Duration	No. Lesion	Size
			(Weeks)		(mm)
0–5	1473	1625	10.32^a^	1.70	13.23 ^b^
	(47.55%)	(52.45%)			
6–10	1593	1871	6.79^c^	1.71	12.03 ^a^
	(45.99%)	(54.04%)			
11–15	984	1240	8.96^b^	2.02	13.30 ^b^
	(44.24%)	(55.76%)			

Superscript letters denote significant (p≤0.05) differences among the age groups.

### Areas of Involvement of Pediatric Cutaneous Leishmaniasis Patients

**Fig 1:**
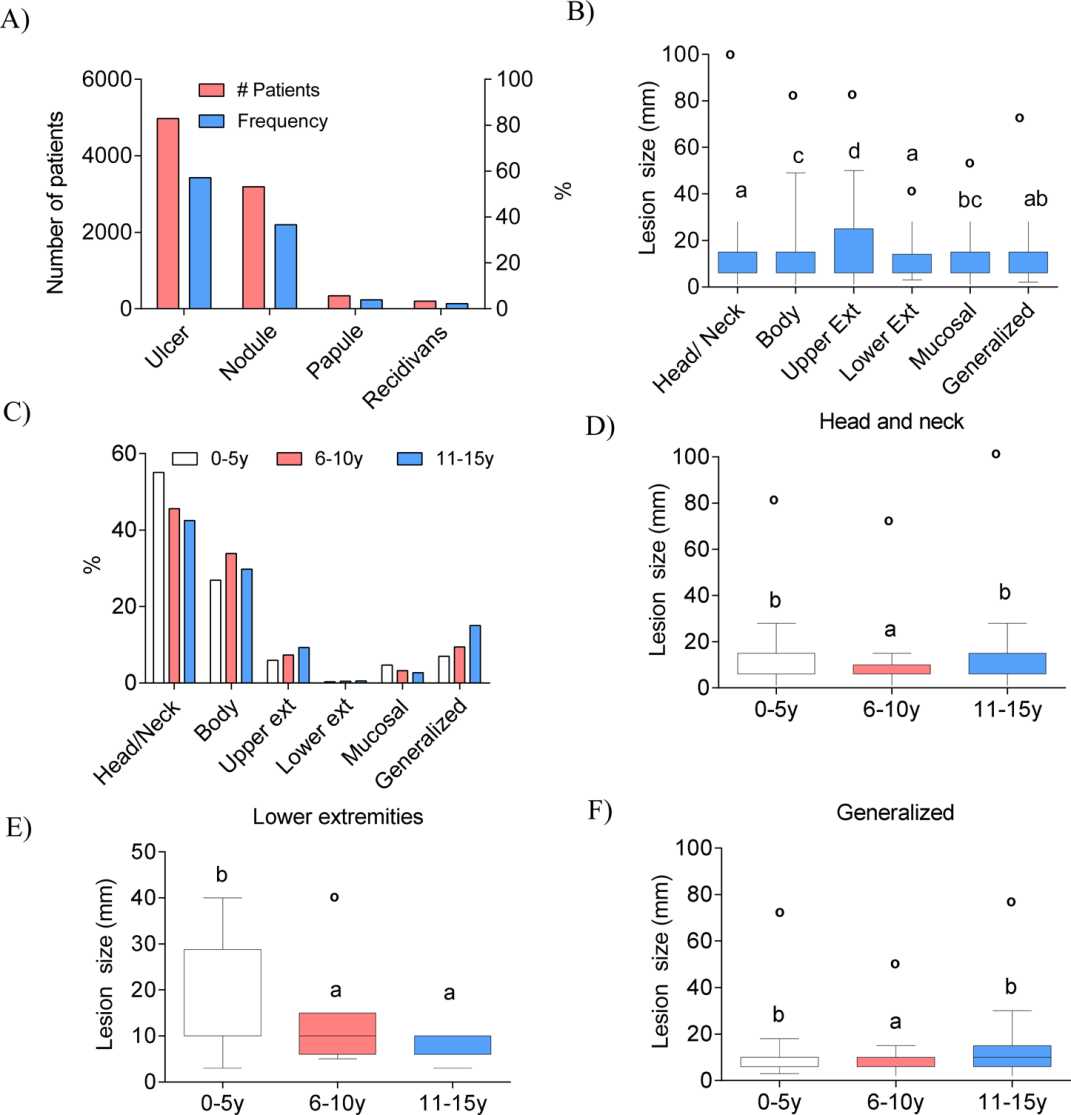
Characteristics of CL lesions in pediatric patients. A) Frequency and number of pediatric patients according to lesion localization. B) Comparison of lesion size in different localizations (p<0.05). C) Frequency of lesion localization classified into the three different age groups. D-F) Differences in lesion size in head and neck, lower extremities and generalized among groups were analyzed using Kruskal-Wallis test followed by posthoc comparison with Dunn-Boneferroni test. Superscript letters denote significant (p<0.05) differences between groups and circles represent outliers.

The area most involved in the lesions of PCL patients was the head and neck region at a rate of 47·8% (n = 4196). The second most involved area was body at a rate of 30·1% (n = 2648). Other areas of involvement were the lower extremity at 0·4% (n = 38), upper extremity at 7·3% (n = 642), mucosa at 3·6% (n = 315) and generalized at 9·9% (n = 874) (involvement of 2 or more areas) (Fig 1A). Also lesion size was different accordingly to the location being upper and lower extremities the sties with larger lesions (Fig 1B). Interestingly, the 0–5 year old group showed more lesions in the head/neck and mucosal regions compared with other groups. Body lesions were most common in the 6–10 year old group. Finally, 11–15 year old group presented less head/neck and mucosal lesions compared with other groups, although this group presented more lesions in the upper extremity and generalized locations (Fig 1C). The lesion size was different between groups in head and neck, lower extremities and generalized (Fig 1D–1F). The 0–5 year old and 11–15 year old groups presented larger lesions in head and neck. In lower extremities 0-5y children presented larger lesions and generalized lesions were larger in both 0-5y and 11-15y groups. The remaining sites of infection were not different in size among groups.

### Clinical Forms of Lesions in Pediatric Cutaneous Leishmaniasis Patients

**Fig 2:**
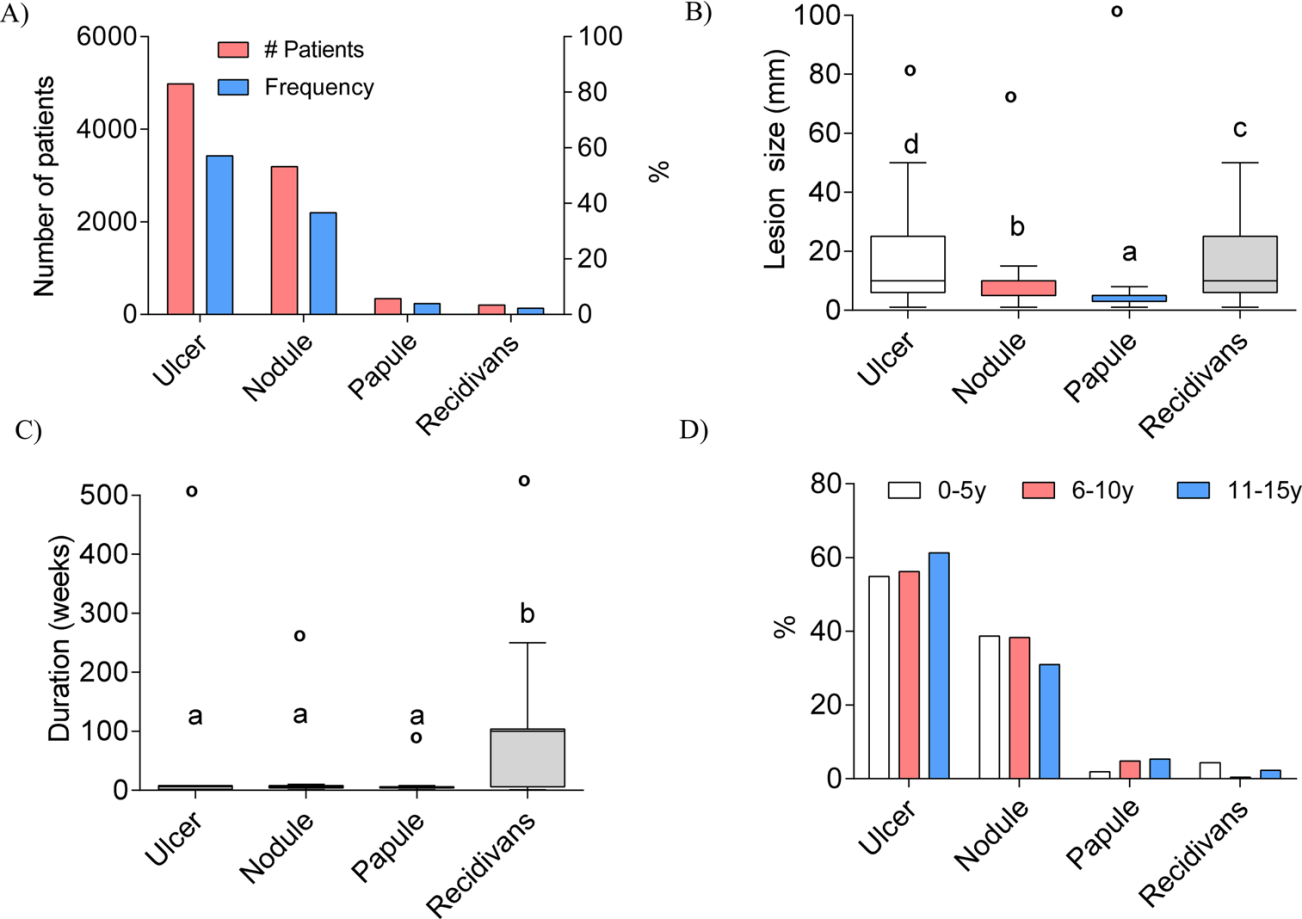
Clinical forms of cutaneous leishmaniasis lesions in pediatric patients. A) Number and frequency of patients presenting with different forms of lesions during cutaneous leishmaniasis. B) Differences in lesion size and C) duration of the disease among the four lesion types. D) Distribution in the frequency of lesion types stratified into three different age groups. Differences in lesion size and disease duration among the lesion types were analyzed using Kruskal-Wallis test followed by posthoc comparison with Dunn-Boneferroni test. Superscript letters denote significant (p<0.05) differences between groups and circles represent outliers.

The most common clinical form of lesions determined in PCL patients was ulcer at 57·14% (n = 4983), followed by nodules at 36·62% (n = 3193), papules at 3·93% (n = 343) and recidivans at 2·29% (n = 200) (Fig 2A). The larger lesions where ulcers and recidivans whereas the smallest lesions were papules (Fig 2B). As expected recidivans were the lesions having the longest duration. However, no significant difference in duration was observed between ulcer nodule and papule (Fig 2C). Ulcers were most common in the 11-15-year-old group, although they presented less nodular lesions. The frequency of ulcers and nodules was similar between 0-5-year-old and 6-10-year-old groups. However, the 0-5-year-old group presented less papules. Recidivans were more common in 0-5-year-old and 11-15-year-old groups. (Fig 2D).

### Response to Intralesional and Systemic Pentavalent Antimonial Therapy in Pediatric Cutaneous Leishmaniasis Patients

During this study there were two types of treatment for cutaneous leishmaniasis. Systemic pentavalent antimonial therapy (intramuscular) was applied to 856 PCL patients (9·74% of the total PCL patients), while intralesional pentavalent antimonial therapy was applied to 6848 PCL patients (77·94% of the total). One thousand and eighty-two patients (12·32% of the total) refused any form of treatment.

**Fig 3:**
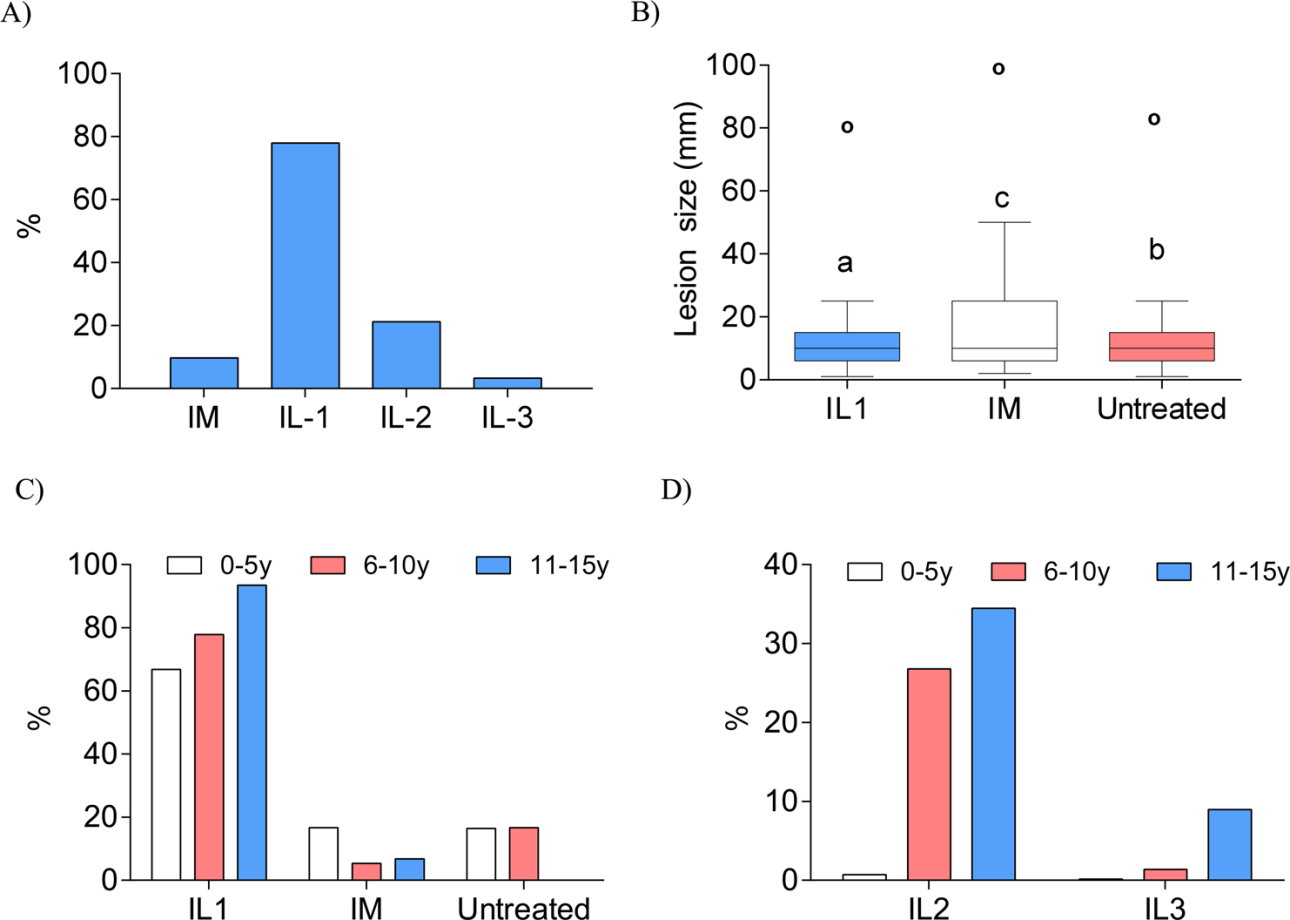
Antimonial therapy of PCL patients. A) Percentage of pediatric CL patients that received intramuscular or intralesional treatments. B) Differences in lesion size among patients who were untreated, received intramuscular, or intralesional antimony treatment. C) Percentage distribution of treatment type administered to patients classified into the three different age groups. D) Percentage distribution of non-responding PCL patients who received a second (IL2) or third cycle (IL3) of intralesional treatment classified into the three different age groups. IL-1: first cycle intralesional treatment, IL-2: second cycle intralesional treatment, IL-3: third cycle intralesional treatment, IM: intramuscular treatment. Differences in lesion size among treatments were analyzed using Kruskal-Wallis test followed by posthoc comparison with Dunn-Boneferroni test. Superscript letters denote significant (p<0.05) differences between groups and circles represent outliers. IM: intramuscular, IL-1 Intralesional cycle one. IL-2 Intralesional cycle two. IL-3 Intralesional cycle three.

Patients that did not heal with the intralesional treatment in the first cycle often received a second or even third cycle of intralesional treatment. Of the PCL patients who received intralesional pentavalent antimonial therapy, 78·79% (n = 5393) received only one cycle (total of 8 injections) of treatment. This left 21·2% of the patients (n = 1455) who required a second cycle of intralesional pentavalent antimonial injection. From the second cycle, only 3·32% of patients (n = 227) received a third cycle (Fig 2A). Patients that received intramuscular treatment presented larger lesions compared to patients that received intralesional treatment or those that were untreated (Fig 3B). Interestingly, when analyzed by groups, the 0-5-year-old group was the least frequent group in which the first intralesional treatment cycle was administered. However, this group also received intramuscular treatment most frequently. Also the 0-5-year-old group received less frequently second and third intralesional treatment cycle. Conversely, while intralesional treatment was more common (93·53%) in the 11-15-year-old group, this group presented the highest frequencies of first cycle (32%) and second cycle (8%) intralesional treatment non-responders. Finally, intralesional treatment was administered in 77% of the patients in the 6-10-year-old group. Interestingly, in this age group, most of the first cycle intralesional treatment non-responders resolved their lesions after the second treatment cycle, while the remaining 1% still required a third treatment cycle (Fig 2C and 2D).

### Cutaneous Leishmaniasis in Pediatric Patients from 1998 to May 2014 in Sanliurfa

**Fig 4:**
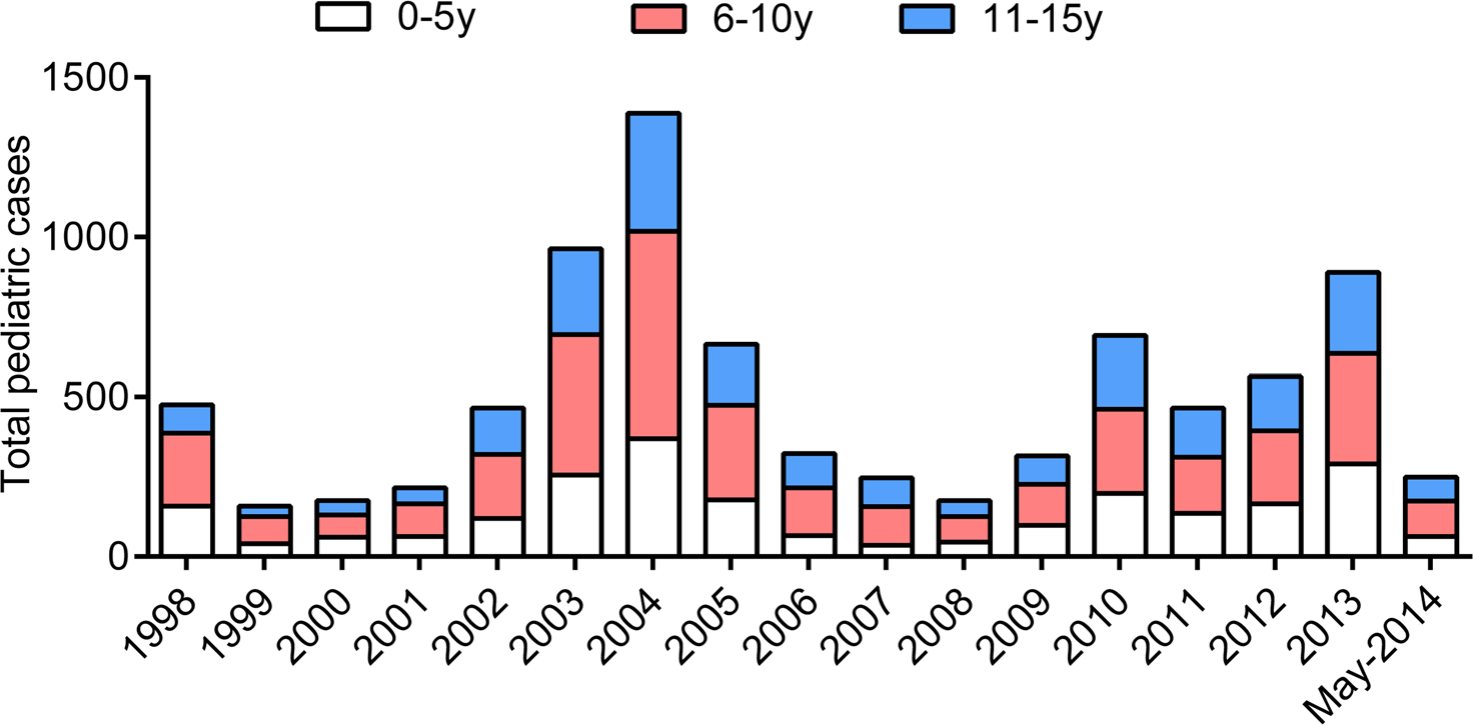
Annual cases of pediatric cutaneous leishmaniasis reported at the Dermatology Clinic of Harran University Medical Faculty and the Oriental Boil Centre of the Sanliurfa Public Health Directorate, Sanliurfa, Turkey from 1998-May 2014. Cases are classified according to the three different age groups.

The last few years have reported increasing cases of CL in Turkey [7]. We determined whether pediatric patients also showed increased numbers of CL cases (Fig 4). Of 8786 pediatric patients, 3098 (35.26%), 3464 (39.43%), and 224 (25.31%) were 0–5, 6-10-11-15 age groups, respectively. We found that total CL cases in pediatric patients peaked in 1998, 2002–2005 and 2010–2013. In all the years considered in this report, the frequency of 6–10 years old patients was higher than the other groups.

## Discussion

Old World CL is seen widespread throughout several countries of the Mediterranean region, including Turkey. The vast majority of CL patients in Turkey have been determined to be in the south-east of the country, particularly in the province of Şanlıurfa (13). In this study we show that CL disease has increased in pediatric patients in the last four years. However, as shown by our current study, the highest incidence was seen in the 6–10 year old age group, and when compared to children, the incidence of CL is very low in the elderly [4,6]. The most likely reason for the greater incidence in children is that children are exposed to the parasite at an early age, and unlike adults, children have not been exposed to leishmaniasis and hence lack immunity to CL.

Diagnosis is generally more difficult in PCL patients than in adults. As PCL patients are often diagnosed with impetigo, prurigo or folliculitis and may receive unnecessary treatment, the clinical form of the disease could become completely changed [20]. Particularly in endemic regions, PCL can be easily diagnosed with clinical examination and a simple smear test. Physicians working in endemic regions in particular must be alert to this disease, especially due to the rising number of CL cases in the last three years in Sanliurfa.

Attempts at different paramedical treatments before the patient consults a doctor may affect the severity and duration of the disease, diameter of the lesions and changes in the characteristics of the lesions. In the current study, the diameter of the lesions in the PCL patients was determined as 12.77±0.11 mm and the number of lesions as 1.76±0.01. The mean diameter of the lesions in the current study was in parallel with the mean lesion diameter and duration of disease reported in PCL patients in other countries [1,10,21–23].

Incorrect diagnosis and treatment of PCL disease could also result in an atypical appearance of lesions, affecting disease duration and resulting in unwanted permanent scars during the healing process. In a study of 160 PCL patients in Tunisia, mean duration of the disease was reported as 12·71 weeks [1]. In the current study, the mean duration of the disease was 8·58 weeks. However, when classified according to age groups, 6–10 year old patients resolved the infection faster, even though this group was more often infected with CL. In contrast 0–5 year old patients took the longest time to resolve the infection. This could be associated with the fact that this group presented recidivans form more often. Also, younger children lack a more mature immune system relative to older children. In CL, lesions are more often seen on uncovered parts of the body as these are the areas that are accessible to the sand-fly. In our current study, a higher rate of PCL lesions was determined on open areas of the body such as the head and neck and the body. These results on areas of the body involved in PCL disease are supported by previous clinical studies [1,10,21–24]. Interestingly, the localization of the lesions varied among groups. In the 0–5 year old group, lesions were more common in the head/neck and mucosal regions. Generalized and upper extremity lesions were more common in 11–15 year old group.

Lesions caused by CL generally start as a small papule or may occur in a papulo-nodular form. When treatment is not applied, lesions grow in diameter and become ulcerated. Sometimes yellowish-red papulo-nodules may be observed on a healed CL scar or around it, which is known as the recidivans form. Patients who do not reside in an endemic region for CL disease may find it extremely difficult to diagnose the recidivans form. In the current study, the most common lesion forms were ulcers and nodules (57·14% and 36·62% respectively). Recidivans form was only determined in 2·29% of the PCL patients. In a study by *Talari et*. *al*., the ulcerated form of lesions was determined in 60·7% of PCL patients and the nodular form in 27·4% [4]. Interestingly, our study showed that recidivans was more common in 0–5 year old group (4.34%) followed by 11–15 year old group (2.31%). These data suggest that a follow up would be necessary for CL patients from 0–5 years old especially for the occurrence of recidivans.

Pentavalent antimonials are used in Turkey for both pediatric and adult CL patients as an effective and safe treatment method and this is often applied in the intralesional form [25]. Interestingly intramuscular injections of pentavalent antimonial were associated with larger lesion size. On the other hand, 78·79% of the PCL patients received only one cycle of intralesional treatment, suggesting that most of the patients responded to the treatment. This is in line with the report by *Zaraa et*. *al*. where they found 86·25% efficacy of intralesional antimonial treatment in PCL patients [1]. We cannot discard however that some patients may have not returned to receive a second cycle or third cycle. In other studies, intralesional antimonial therapy has been reported to be effective in PCL patients [10,22,24–26]. When intralesional antimonial therapy is applied correctly and regularly, it remains the gold standard of treatment for PCL patients. In our study we found that in the 0-5-year-old group, intralesional therapy was applied only to 66% of the patients, while 16% received intramuscular therapy. This could be explained by the fact that IL lesions require repeated administration of the drug, which is painful and may result in poor patient compliance. For pediatric patients, the parents and/or doctors may prefer a single intramuscular dose (16·72%), while others may refuse treatment (16%). However, systemic administration of antimonials is known to be toxic, [26] and it is not known if it could affect childhood development. Interestingly, the 0–5 year old group most frequently received one intralesional cycle, probably because they responded more efficiently than the other groups to intralesional treatment, with no need for a second cycle. Therefore, intralesional treatment of 0–5 year old patients may be considered as a preferential form of treatment for CL, so as to avoid the toxicity associated with systemic administration of antimonials. Most of the children in the 11–15 year old group received intralesional treatment, although there were more non-responders in this group who needed a second cycle (34·4%) and a third cycle (8·9%) of treatment. Some pediatric patients between 6–10 years old needed a second cycle (26·79%) but only very few needed a third cycle (1·37%) of treatment. These data should help in the determination and prognosis of the more appropriate form of treatment for CL depending on the age of the children.

In conclusion, CL affects mainly children and is more common in the 6–10 year old range. Interestingly, the categorization of pediatric patients revealed different clinico-epidemiological characteristics of CL including duration of disease, size of lesion, and lesion localization depending on the age of the children. Importantly response to antimonial therapy appears to be dependent on age. The results from this study can help to improve clinical care and treatment of pediatric CL patients in Turkey.
